# AI‐Enhanced Semantic Feature Norms for 786 Concepts

**DOI:** 10.1111/tops.70037

**Published:** 2025-12-30

**Authors:** Siddharth Suresh, Kushin Mukherjee, Tyler Giallanza, Xizheng Yu, Mia Patil, Jonathan D. Cohen, Timothy T. Rogers

**Affiliations:** ^1^ Department of Psychology University of Wisconsin–Madison; ^2^ Department of Computer Sciences University of Wisconsin–Madison; ^3^ Department of Psychology Stanford University; ^4^ Department of Psychology Princeton University; ^5^ Department of Computer Science Brown University; ^6^ Princeton Neuroscience Institute Princeton University

**Keywords:** Semantic knowledge, Feature listing, Large language models, Similarity judgments

## Abstract

Semantic feature norms have been foundational in the study of human conceptual knowledge, yet traditional methods face trade‐offs between concept/feature coverage and verifiability of quality due to the labor‐intensive nature of norming studies. Here, we introduce a novel approach that augments a dataset of human‐generated feature norms with responses from large language models (LLMs) while verifying the quality of norms against reliable human judgments. We find that our AI‐enhanced feature norm dataset, NOVA: **N**orms **O**ptimized **V**ia **A**I, shows much higher feature density and overlap among concepts while outperforming a comparable human‐only norm dataset and word‐embedding models in predicting people's semantic similarity judgments. Taken together, we demonstrate that human conceptual knowledge is richer than captured in previous norm datasets and show that, with proper validation, LLMs can serve as powerful tools for cognitive science research.

## Abstract 2

We introduce NOVA, an AI‐enhanced semantic feature norm dataset for 786 concepts. By augmenting human‐generated features with LLM responses validated against human judgments, NOVA reveals richer semantic structure than traditional norms. The dataset better predicts human similarity judgments, suggesting previous methods underestimated the richness of human conceptual knowledge.

## 1 Introduction

The study of human conceptual knowledge has relied on semantic feature norms—representations of concepts in terms of their associated features—since their introduction by Rosch in the 1970s (Rosch, 1975). Norming studies present participants with a set of concepts and, for each, ask them to list as many characteristic properties as they can. Aggregating features across items and participants creates semantic vectors the elements of which correspond to the elicited features and the entries of which indicate whether people regularly judge the concept to possess the corresponding property. Proximity between two such feature vectors relates systematically to their perceived semantic relatedness—thus, lions and tigers are viewed as similar kinds of things because they have many overlapping and few distinguishing properties. Norming datasets collected over the years (Buchanan et al., 2019; Devereux, Tyler, Geertzen, & Randall, 2014; Dilkina et al., 2008; Hansen & Hebart, 2022; McRae et al., 2005; Ruts et al., 2004) have helped to answer questions about the organization of semantic memory (Collins & Loftus, 1975; Ashcraft, 1978), its degradation in semantic disorders (Cree & McRae, 2003; Farah & McClelland, 2013; Garrard et al., 2001; Rogers & McClelland, 2004), its relationship to control (Giallanza et al., 2024), and its neural bases (Cox et al., 2024; Clarke & Tyler, 2014) (see Kumar (2021) for a review).

Taking a broader view across these studies, a key role semantic vectors derived from norming studies play in cognitive science is in defining a key representational target that neurocognitive theories of semantic memory must meet. The general approach for identifying the neural loci for a construct under study (e.g., semantic memory) has often involved using a variety of analytic techniques to map variation of neural population activity (measured using, e.g., functional Magnetic Resonance Imaging (fMRI), Electroencephalography (EEG), MEG, Electrocorticography (ECoG)) to some form of experimenter‐defined targets that represents properties of the stimulus (Frisby et al., 2023). These targets range from one‐hot codes representing different categories of stimuli (Norman et al., 2006; O'Toole et al., 2007) to semantic vectors (Pereira et al., 2018) representing properties of stimuli. Semantic vectors derived from semantic norms constitute a particularly useful representation since the individual dimensions of the vectors are interpretable as nameable features and the fact that all concepts share the same feature space allows researchers to capture potentially rich patterns of cross‐domain structure (e.g., between places and objects) that other representations might not. This is significant insofar as we want our theories of the operations carried out by the brain to be able to capture this form of cross‐domain structure. Thus, while a large‐scale semantic norm dataset for a diverse range of concepts is desirable, the construction of such a dataset has been challenging for a few reasons.

Semantic norming requires extensive human labor both in data collection and curation/post‐processing. Prior studies have met this challenge in different ways, each requiring some degree of compromise as elaborated below. Other recent work has sought alternatives to human feature norms by making the use of natural language processing technologies, including word embeddings from methods such as word2vec and GloVe (Mikolov et al., 2013; Pennington et al., 2014), as well as feature norms generated artificially by large language models (LLMs) (Hansen & Hebart, 2022). However, word embeddings fail to capture the semantic structure perceived by humans as effectively as feature norms, and their dimensions lack the transparent interpretability of feature‐based representations, at least for concrete objects (Suresh et al., 2023, 2023). LLMs are a promising avenue for accelerating cognitive science research given that not only can they respond to queries posed in natural language (similar to how human participants are probed), but have also been shown to approximate various facets of human cognition (Aher et al., 2023; Binz & Schulz, 2023; Bubeck et al., 2023; Marjieh et al., 2022; Webb et al., 2023). However, they are not without their limitations. For our task of interest, while they can generate super‐human lists of features that go far beyond what a typical person might know, this very fact might make them nonrepresentative of human knowledge. The responses they generate should also not be taken to be reliable without further verification given that they frequently confabulate properties that are untrue (the well‐documented “hallucination problem” (Huang et al., 2024)).

The current work seeks a middle way between human‐only and machine‐only norm generation. We first crowd‐sourced feature lists for a modestly large and representative set of 786 concrete object concepts thus ensuring that the features included in the set are those that human participants discern. Next, we implemented a feature verification procedure first used by Ruts et al. (2004) on a set of Dutch semantic norms (De Deyne et al., 2008) using a subset of our concept‐feature pairs. Unlike Ruts et al. (2004), who applied their verification procedure within broad semantic categories (e.g., “plants,” “animals”), we applied the procedure across concepts regardless of their broad semantic category membership ensuring that we captured deeper cross‐domain structure. We leverage the ability of state‐of‐the‐art language models like OpenAI's GPT‐4o (Achiam et al., 2023) to perform this verification procedure, and first validated their performance against ground‐truth human judgments and scaled up the procedure to a larger set of concept‐feature pairs once we were confident about the best configuration of models.

The final result of this procedure was a novel *AI‐enhanced* set of semantic feature norms—NOVA: **N**orms **O**ptimized **V**ia **A**I. Using NOVA, we illustrate remarkable differences between human‐only and AI‐enhanced norm sets, then report empirical studies designed to assess whether the AI‐enhanced norms capture human‐perceived semantic structure better than do human‐only norms or “out‐of‐the‐box” word embeddings.

## 2 Study I: Building NOVA

**Fig. 1:**
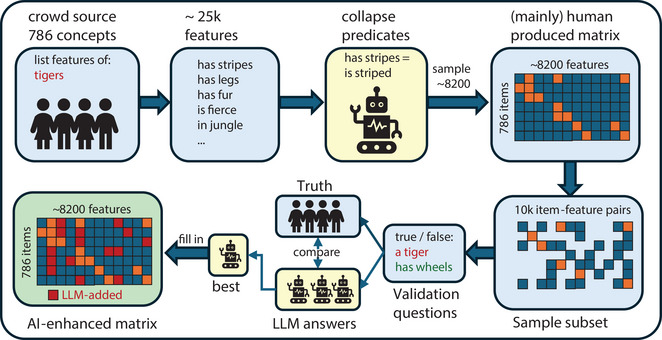
A schematic representation of our workflow. Features were initially crowd‐sourced for 786 concepts, forming a human‐generated matrix. A subset of 10,000 concept‐feature pairs underwent validation via human judgments. LLM responses were compared to these human judgments to determine the best‐performing strategy. Using this method, LLMs completed the matrix for all 8200 selected features, forming the AI‐augmented matrix.

Human feature‐norming studies involve up to four steps, each requiring significant effort and thus subject to constraints that can limit the resulting data. Here, we consider each step, limitations faced by prior studies, and the approach taken in the current work. The overall workflow for our approach is shown in Fig. 1.

*Concept selection*. The structure appearing in a given dataset depends on the concepts included. Early norms used in semantic memory studies focused on hierarchically structured, easily nameable concepts (e.g., animals, plants), often excluding typical examples (e.g., robins, sparrows) in favor of atypical ones easier to name (e.g., penguins, ostriches) and omitting concepts that do not fit neatly into these hierarchies. To improve representativeness, we included all 565 concepts from the Ecoset dataset (Mehrer et al., 2021), which comprises frequent, unambiguous basic‐level concrete object names, along with items from the McRae (McRae et al., 2005) and Leuven (De Deyne et al., 2008) norms. We also added superordinate categories (e.g., animal, vehicle) and higher‐frequency subordinate names (e.g., robin, trout) to better capture domain substructure. The final set comprised **786** concrete object concepts.

*Feature elicitation*. We elicited features from participants on Amazon Mechanical Turk using procedures described below.

*Feature reduction*. Norming studies typically yield a large set of unique features, most appearing in a single concept. To manage this complexity, researchers often consolidate distinct yet semantically related properties— for example, if *is hairy* and *is furry* are used by different participants to describe a “coconut,” these features may be deemed as equivalent, creating a single feature that overlaps for concepts possessing both *is hairy* (e.g., ape) and *is furry* (e.g., rabbit). While this process simplifies the feature space and enhances conceptual similarity across items, it is labor‐intensive and relies on subjective human judgments. We instead performed a minimal feature collapse by using GPT‐3 to extract phrase embeddings of featural descriptions (e.g., *has a furry outer layer*), then clustering these and merging only highly similar clusters. This approach collapsed phrases with variable wording but near‐identical semantic content (e.g., *has a furry outer layer*, *is furry*, and *feels furry*) while still distinguishing close synonyms (e.g., *is hairy* vs. *is furry*). This step reduced the initial ∼25k raw features to ∼20k features, from which we randomly sampled ∼8200 features for subsequent analysis.

*Feature verification*. The features that participants generate in the elicitation phase typically constitute a fraction of what they actually know. For this reason, some norming studies conduct a *feature verification* step where human participants consider every concept/feature pair and judge whether the feature is true of the concept (De Deyne et al., 2008; Dilkina et al., 2008). This step greatly enriches the structure encoded in the norms. For instance, most participants list the feature *has a long neck* for giraffes and swans but for few other items. Yet, when asked, most participants agree that *has a long neck* is true of items as varied as a duck, a beer bottle, and a cello. Thus, the verification phase surfaces latent knowledge that participants do not generate spontaneously. Since the number of concept/feature pairs grows exponentially, this is by far the most labor‐intensive part of the process and prior studies have either employed a relatively modest set of concepts and features (Dilkina et al., 2008) or have limited verification only to specific semantic domains (De Deyne et al., 2008). We leveraged LLMs to conduct the feature‐verification phase—first comparing different models and strategies in their ability to capture human judgments on a randomly sampled set of concept‐feature pairs, then using the most successful strategy to verify all ∼6.5M concept/feature pairs, producing an AI‐enhanced norm set.

### 2.1 Methods

#### 2.1.1 Human feature elicitation

This phase provided human‐elicited data for all concepts in the set, providing the raw features from which human‐only and AI‐enhanced norms were derived.

*Participants*. Fifty participants were recruited through Amazon Mechanical Turk and were compensated $4 for the task, which would require 20 min to complete. The study was approved by the Princeton Internal Review Board, IRB Protocol 6079.

*Stimuli and procedure*. Stimuli were 786 concrete object nouns. Using a web‐based interface, each participant viewed up to 75 different words in randomized order, and for each typed in as many different features as they could generate. The instructions emphasized generating various types of features, including physical/perceptual features (appearance, smell), functional features (uses, contexts), and other characteristics. Participants were asked to format their responses as individual features per line using standardized phrasing (e.g., “has ears” rather than “a dog is an animal that has ears”).

#### 2.1.2 Human feature verification

This phase had human participants verify ∼10k concept‐feature pairs, providing an empirical basis for evaluating the performance of different AI‐aided approaches to feature verification.

*Participants*. Five hundred and fifty‐six participants were recruited through Amazon Mechanical Turk and compensated $1.40 for a 5–8 min task. Participants were allowed to complete multiple sessions contingent upon maintaining satisfactory performance.

*Stimuli and procedure*. The stimuli were concept‐property pairs sampled randomly from results of the feature‐elicitation task. Data were collected through an online interface. Each trial paired one concept (e.g., “alligator”) with one feature randomly sampled from the full set. The sampled feature could come from any domain or item—for alligator, it could be something reasonable (e.g., “has legs”), something clearly false (e.g., “has wheels”), or something uncertain (e.g., “has ears”). For each pair, participants judged whether the property is true of the item by pressing a keyboard button. The instructions emphasized that subjective properties should be evaluated based on common consensus (e.g., “cute” for “dog”), and properties that were sometimes true should be marked as true (e.g., “brown” for “dog”). Each participant made about 110 judgments, and we collected five or more judgments on each of 10,545 unique pairs. Participants could skip unfamiliar concepts or nonsensical properties by pressing the space bar, with skipped items replaced to maintain the required number of judgments.

#### 2.1.3 AI‐enhanced feature verification

Our ultimate goal was to use LLMs to complete the feature‐verification step for all possible concept/property pairs. Since there are millions of possible pairs, we first considered how well each of several different models and prompting strategies could capture real human judgments on the items collected in the human feature‐verification study. In these data, participants showed different opinions for about 40% of the items—thus, either opinion expressed by an LLM would agree with at least one human participant for these items. We, therefore, selected the 6,122 concept‐feature pairs for which all participants made the same decision (either all yes or all no), and used these decisions as a ground‐truth for evaluating LLM performance.

*Model suite*. We primarily focused on performant open‐sourced language models because these are accessible to other researchers for replication purposes and relatively more affordable to access. We included models that have open weights, are generally high‐scoring on standard LLM benchmarks (Hendrycks et al., 2020), and can be run on consumer‐grade hardware. Specifically, we evaluated three models from Meta's Llama family (Llama3, Llama3.1, and Llama3.2) (Dubey et al., 2024), Microsoft's Phi‐4 (Abdin et al., 2024), Ai2's Olmo2 (OLMo et al., 2024), and Google's Gemma2 (Team et al., 2024) and Flan‐T5 (Wei et al., 2021). We evaluated all models at full bfloat16 precision on a Nvidia H100 GPU. For comparison to a state‐of‐the‐art closed model, we also evaluated GPT‐4o via its API.

*Evaluation protocol*. We prompted all models using the following general prompt ‐

In one word True or False, answer the following question:
Is the property [x] true for [y]? Answer:
…where x was a feature and y was a concept with the square brackets included in the prompt. We ran two prompting experiments: (1) a zero‐shot experiment providing the models with just the question above as input, and (2) a two‐shot experiment providing the models with two example feature‐concept pairs, one true and one false, to potentially improve the models' ability to perform the task via in‐context learning (Brown et al., 2020). We used the same two examples for all prompts.

*Post‐processing*. To extract meaningful answers from model‐generated text, we first restricted responses to a maximum of five tokens, then conducted a case‐insensitive search of model responses for the strings “True” or “Yes” to indicate a positive response, and “False” or “No” to indicate a negative response. In rare cases where no match was found, we set the model response to “False.”

### 2.2 Results

To measure how closely LLM responses aligned with unanimous human judgments for the 6,122 feature‐concept pairs, we adopted a signal detection approach, treating human responses as the true signal and model responses as guesses. Where humans agreed the property was true of the concept, model guesses were scored as hits if they concurred and misses otherwise. Where humans agreed the property was not true of the concept, model guesses were scored as correct rejections if they concurred and false alarms otherwise. From these counts, we computed hit rates and false alarms rates, then converted these to the $d^{\prime }$ measure of signal discrimination.

**Fig. 2:**
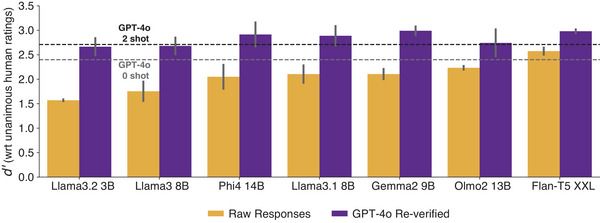
Models' ability to reliably predict human feature‐concept ratings measured as $d^{\prime }$ using raw responses (orange) and responses reverified using GPT‐4o. Bar heights show mean $d^{\prime }$ across the 0‐shot and 2‐shot experiments. Gray and black dashed lines correspond to GPT‐4o's performance in the 0‐shot and 2‐shot settings, respectively. Error bars correspond to bootstrapped 95% confidence intervals.

The average $d^{\prime }$ for both zero and two‐shot conditions can be seen in Fig. 2 (yellow bars). Two‐shot GPT‐4o outperformed all open‐sourced models, which varied in their match to human responses. Two‐shot Flan‐T5 XXL performed best among open‐sourced models and better than the zero‐shot GPT‐4o. Flan‐T5's lower $d^{\prime }$ relative to GPT‐4o was driven by a propensity to respond with “true” to many queries, buoying its hit rate but also increasing its false‐positive rate. To preserve the benefits of GPT‐4o without incurring a prohibitive cost, we next considered a “reverification” approach in which the “true” responses generated by a given open‐source model were subsequently reverified by GPT‐4o, retaining the “true” value only if both models agreed. The results are shown as purple bars in Fig. 2. Reverification improved performance for all models, surpassing GPT‐4o alone. Flan‐T5 XXL remained a top model, closely matched by Gemma2 9B. Given the strong baseline performance of Flan‐T5, we chose this model with GPT‐4o reverification to fill out the full semantic feature matrix.

**Fig. 3:**
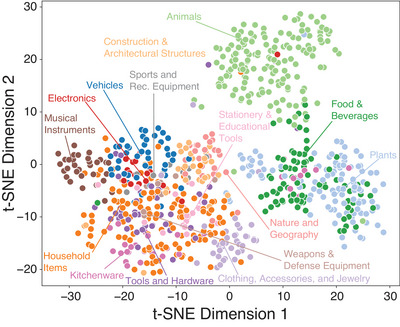
t‐stochastic neighbor embeddings of the semantic vectors for each of 786 concepts derived from the final verified matrix. Category labels were generated by combining higher‐order labels from existing norm datasets and LLM‐suggested categories from GPT‐4o.

**Using Flan‐T5 and GPT‐4o to impute the AI‐enhanced matrix**. In the human‐only matrix, entry [i,j] has a value of 1 wherever a participant produced feature j for concept i and a 0 in all other entries. For every 0 in this matrix, we prompted Flan‐T5 XXL to decide whether the corresponding property is / is not true of the corresponding concept. Where the model decided “not true,” the zero value was retained in the matrix. Where the model decided “true” (534,010 out of 6,436,554 possible pairs), we prompted GPT‐4o with the same pair to reverify the answer. If GPT‐4o agreed the property was true, the cell value was replaced with 1, otherwise the 0 value was retained. This procedure yielded the final **AI‐enhanced** norms matrix. Fig. 3 shows t‐SNE‐based embeddings of all concepts from this matrix.

**Fig. 4:**
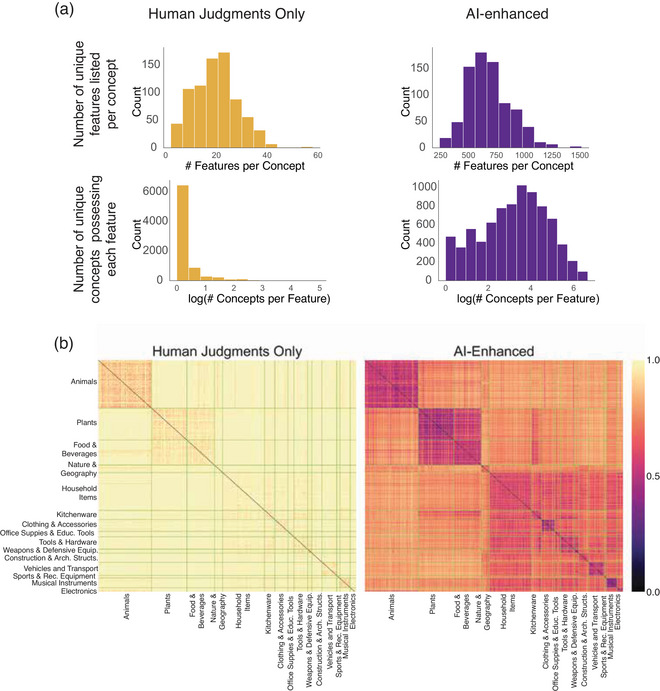
(a) Counts of valid features per concept and number of concepts that share common features for the reduced human‐generated matrix (top row) and AI‐enhanced norm matrix (bottom row). (b) Pairwise cosine dissimilarity matrices based on the reduced human‐generated norm matrix (left) and AI‐enhanced norm matrix (right).

The AI‐enhanced matrix differed remarkably from the human‐only matrix in its feature density. While the human matrix has about 20 features per concept on average, the AI‐enhanced matrix has about *700* (Fig. 4a), and while the majority (78%) of features in the human‐only matrix are true of just one concept, this is true of just 5% of features in the AI‐enhanced matrix. The increased feature density produces much more richly structured similarity relations, as shown by the heat plot of pairwise distances between concepts in Fig. 4b. While some of this difference may be attributable to false‐positives in the AI‐enhanced dataset, the comparison to human judgments suggests that the LLM verification strategy is quite good at discriminating true positives from true negatives ($d^{\prime }>3.0$). Thus, the result suggests that human knowledge about features of concepts may be considerably richer than prior norming studies have suggested.

## 3 Study 2: Using the new norms dataset to predict human semantic judgments

To assess whether the AI‐enhanced norms in NOVA capture information about semantic structure beyond human‐only norms or other approaches, we compared different approaches in their ability to predict human behavior in a triadic similarity judgment task (Hebart et al., 2023; Jamieson et al., 2015; Sievert et al., 2023). In this task, participants must decide which of two option concepts is semantically more similar to a target concept. A candidate semantic embedding can “predict” human decisions by selecting whichever option word lies closer to the target word in the embedding space. We can assess the quality of the embedding by comparing how often the predicted response agrees with actual human decisions. In this study, we compared the predictions of NOVA embeddings to those based on the human‐only feature norms and to those generated by a common word‐embedding approach (FastText).

### 3.1 Methods

**Fig. 5:**
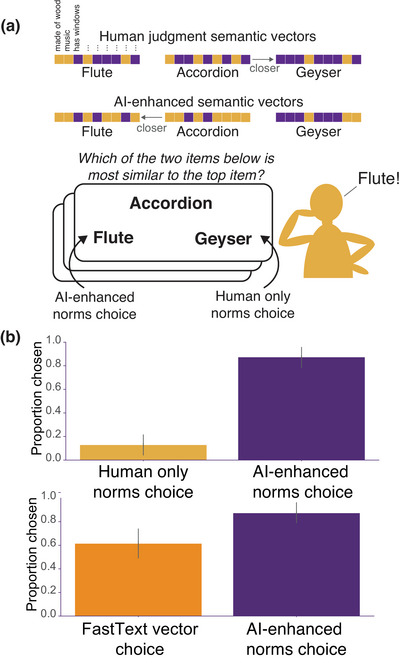
(a) Procedure for generating trials for the triadic judgment experiment and an example trial. (b) Proportion of human responses that aligned with the human matrix (yellow bar) versus the AI‐enhanced matrix (purple bar) and with FastText word embeddings (orange) versus AI‐enhanced semantic vectors (purple) in Experiment 2. Error bars represent standard errors of the means.

We selected triplets designed to maximally discriminate NOVA and human‐only feature norms. Thus, for each trial, one of the option items was closer to the target in the human‐only space, while the other was closer in the AI‐enhanced space (see Fig. 5). We then computed how often the majority‐vote across human participants agreed with the predictions of each embedding (AI‐enhanced, human‐only, FastText). If the AI‐enhanced norms in NOVA contain information irrelevant to human‐perceived semantics, their predictions should agree with human judgments less often than do those of the human‐only norms. Furthermore, if either set of norms simply recapitulates the semantic structure evident in word embeddings, then predictions from the norms should do about as well as predictions from the FastText embeddings.

#### 3.1.1 Generating maximally disagreeing triplets

To generate triplets that maximally differentiated the human‐only and AI‐enhanced norms, we computed cosine dissimilarity matrices for each set (Fig. 4b), Procrustes‐aligned them to minimize disparity, and identified concepts with the largest discrepancies in their distances to other concepts. For example, in NOVA space, “accordion” was closer to “flute” than to “geyser,” while the reverse was true in the human‐only space (Fig. 5a). We constructed 1,424 triplets where the two matrices produced divergent predictions, with each of the 786 concepts serving as the target approximately twice. The critical question was which matrix's predictions would align more closely with human similarity judgments.

*Participants*. Thirty‐one participants were recruited from the UW‐Madison psychology subject pool. Participants completed the task online for course credit. Each participant provided informed consent in compliance with the UW‐Madison IRB.

*Stimuli and procedure*. The stimuli were the set of 1,424 triplets described above. Data were collected online via jsPsych (De Leeuw, 2015). On each trial, a randomly selected triplet was displayed, with participants indicating which of two options was more similar to the target concept using a mouse click. All triplets were judged by each participant.^1^

### 3.2 Results

Human similarity judgments agreed with predictions of the AI‐enhanced norms for 86.20% of triplets, a result unlikely to arise by chance (p< .001, binomial test). Human judgments agreed with predictions of the FastText embeddings on 60.40% of trials: reliably better than chance (p< .001, binomial test), but significantly worse than the AI‐enhanced embeddings (paired t‐test, t(1,423) = 18.37, p< .001). Thus, the richer structure evident in the AI‐enhanced feature norms appears to better express human‐discerned semantic similarity structure than to norms derived from humans alone or from word‐embeddings.

## 4 Discussion

We presented a new approach for generating AI‐enhanced semantic norms along with an accompanying dataset, NOVA. We first conducted controlled experiments evaluating LLM feature verification performance against a reliable subset of human norm judgments, using the results to find an optimal model, prompting strategy, and verification strategy. We then applied the best‐performing approach to generate an AI‐enhanced large‐scale norm dataset spanning over 750 concepts and over 8,000 features. Concepts in the resulting NOVA dataset showed much higher feature density and a greater degree of feature overlap relative to the raw human‐generated matrix. This overlap of features did not come at the cost of category selectivity, with concepts being reasonably organized into meaningful clusters. Finally, we used a triadic comparison task to show that NOVA vectors more accurately predicted human similarity judgments than did vectors based on human norms alone or word embeddings computed from natural language. The result suggests that AI‐enhanced norms express semantic structure more similar to that discerned by human participants.

The development of NOVA opens new opportunities to reveal a more comprehensive neural basis of semantic knowledge than previously documented. Existing efforts to decode semantic representations in the mind and brain (Frisby et al., 2023) have largely depended on categorical labels or semantic vectors derived from either opaque Natural Language Processing (NLP) models or semantic norms that underestimate the diversity of human concepts and the richness of their relationships. As a result, these studies may have underestimated the diversity and complexity of the neural code that supports conceptual knowledge. NOVA provides wider conceptual coverage, has a richer basis of features, and outperforms embedding models like FastText in predicting human semantic similarity judgments, remedying the limitations of traditional decoding targets. With the concurrent development of analytical tools capable of decoding distributed neural codes for properties once thought to be highly localized (Cox & Rogers, 2021; Cox et al., 2024; Oswal et al., 2016), future research in this domain can aim toward a neurocognitive account of semantic knowledge that is capable of capturing the representational richness found in NOVA.

Taken together, our work addresses longstanding limitations in semantic norm generation by creating a dataset that includes a representative set of concepts and features, with AI‐based feature verification validated against human judgments. The feature density of the AI‐enhanced norms reveals semantic similarity structure richer than previous norm datasets, unlocking the potential to better understand both the cognitive and the neural bases of semantic memory (Clarke & Tyler, 2014; Cox et al., 2024; Fernandino et al., 2022; Rogers & McClelland, 2004) and to guide the development of future computational neurocognitive models (Dilkina et al., 2008; Giallanza et al., 2024; Riordan & Jones, 2011; Saxe et al., 2019; Suresh et al., 2024). Lastly, the present work highlights the promise of integrating LLMs into workflows for cognitive science research in a controlled and verifiable manner and provides a replicable framework for future endeavors in this domain (Dillion et al., 2023; Mukherjee et al., 2024, 2023; Suresh et al., 2023; Trott, 2024).

## Data Availability

All code and materials will be available at: https://github.com/Knowledge‐and‐Concepts‐Lab/llm‐norms‐cogsci2025

## Note

1 There was data loss of a few trials for some participants due to technical issues.
